# The Desensitization Gating of the MthK K^+^ Channel Is Governed by Its Cytoplasmic Amino Terminus

**DOI:** 10.1371/journal.pbio.0060223

**Published:** 2008-10-28

**Authors:** Mario Meng-Chiang Kuo, Innokentiy Maslennikov, Brent Molden, Senyon Choe

**Affiliations:** Structural Biology Laboratory, The Salk Institute, La Jolla, California, United States of America; University of Texas, United States of America

## Abstract

The RCK-containing MthK channel undergoes two inactivation processes: activation-coupled desensitization and acid-induced inactivation. The acid inactivation is mediated by the C-terminal RCK domain assembly. Here, we report that the desensitization gating is governed by a desensitization domain (DD) of the cytoplasmic N-terminal 17 residues. Deletion of DD completely removes the desensitization, and the process can be fully restored by a synthetic DD peptide added in trans. Mutagenesis analyses reveal a sequence-specific determinant for desensitization within the initial hydrophobic segment of DD. Proton nuclear magnetic resonance (^1^H NMR) spectroscopy analyses with synthetic peptides and isolated RCK show interactions between the two terminal domains. Additionally, we show that deletion of DD does not affect the acid-induced inactivation, indicating that the two inactivation processes are mutually independent. Our results demonstrate that the short N-terminal DD of MthK functions as a complete moveable module responsible for the desensitization. Its interaction with the C-terminal RCK domain may play a role in the gating process.

## Author Summary

Nerve cells use ion channels, pores in the cell membrane, to send messages in the form of electrical signals between cells. Most ion channels have evolved several elaborate mechanisms that allow the channels to close quickly after opening to prevent wasteful leakage of the electrochemical potential—the currency of neuron communication—across the cell membrane. The process is known as inactivation or desensitization. Previous study on the model RCK-containing MthK K^+^ channel in the enlarged Escherichia coli membrane has shown that this archaeon channel also undergoes desensitization. Using the same method, we demonstrate that the desensitization is indeed an intrinsic molecular property of the MthK protein. We show that a specific region of MthK, the short N terminus of the protein, functions as a structurally independent domain and is entirely responsible for the desensitization gating process. Moreover, we show that this N-terminal domain interacts with the C-terminal RCK domain as part of the desensitization mechanism. This unique desensitization mechanism, by interaction between the two cytoplasmic termini, is distinct from those traditional mechanisms known as N- and C-type inactivation found in many voltage-gated Na^+^ and K^+^ channels or as the desensitization observed in the glutamate receptors. Since the KTN/RCK domain is found in a large number of prokaryotic K^+^ channels and transporters, this unique mechanism may be common to these transport systems for regulating the K^+^ flux through the cell membrane.

## Abstract 3

The N terminus of the ion channel MthK functions as a structurally independent domain and is entirely responsible for the desensitization gating process required for neuron-to-neuron communication.

## Introduction

K^+^ channels are found in almost every free-living organism, with a universally conserved architecture. Typically, a functional K^+^ channel is composed of four copies of a pore-forming subunit of two or six transmembrane (TM) helices with the amino (N)- and carboxyl (C)-termini usually residing in the cytoplasm. These cytoplasmic N- or C-terminal domains can control channel assembly and trafficking, as well as function as a gatekeeper to regulate the access of K^+^ to the ion-conducting pathway [1–5]. A particular form of control by cytoplasmic domain is exemplified by the RCK [6] (also known as KTN [7]) domain found in a large number of prokaryotic K^+^ transport systems, including ion channels and transporters [8], and also in the animal Slo-type K^+^ channels [6]. The crystal structure of the RCK-containing MthK channel (M107I mutant), from the archaeon Methanobacterium thermoautotrophicum, provides a relatively simple model, allowing direct structural, biochemical, and functional correlations to understand the regulatory roles of the RCK domain in K^+^ channels [9].

**Figure 1:**
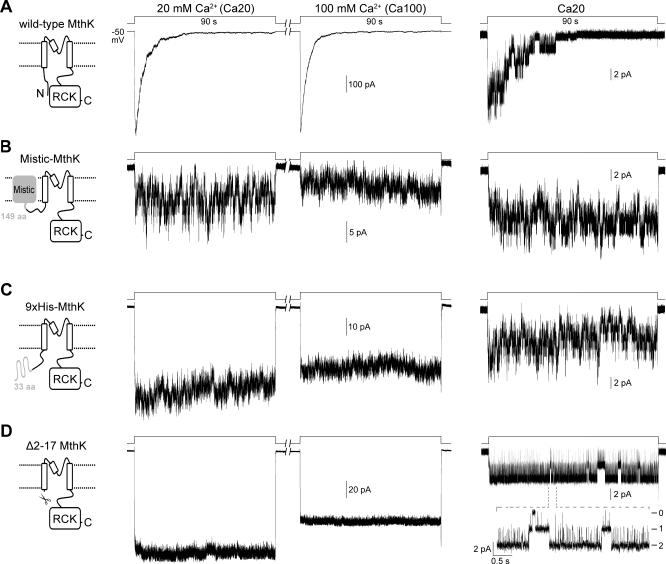
Representative Macro- and Microscopic Traces of Different MthK Constructs Expressed in E. coli Membrane Excised inside-out patches were held at −50 mV. Macroscopic traces (left and middle, from same patches) were recorded from cells with IPTG treatment. Single-channel traces (right) were recorded from cells without IPTG treatment. Channels were activated by stepping the perfusate from EGTA to either Ca20 or Ca100 solution at pH 7.5 using a rapid perfusion system. (A) Traces of wild-type MthK. The macroscopic Ca^2+^-activated current decays in a few seconds after being activated by 20 (left trace) or 100 (middle trace) mM Ca^2+^. The single-exponential time constants for the decays are 5.7 ± 0.6 s and 4.2 ± 1.2 s (*n* = 6), respectively. Right trace, a single-channel trace shows that the channel's open probability decreases during the extended 20 mM Ca^2+^ perfusion. (B) A Mistic protein [16] was fused to the N-terminus of MthK to create the Mistic-MthK chimera (Materials and Methods). The traces show that the chimeric channels remain active during the prolonged Ca^2+^ perfusion. (C) Fusing a nona-histidine-containing peptide (Materials and Method) to the N-terminus of MthK also results in chimeric channels that remain active during the sustained Ca^2+^ perfusion. (D) The entire cytoplasmic N-terminus of MthK was deleted to create the Δ2–17 MthK. Macroscopic traces of the deletional mutant shows that the current does not decay in either 20 or 100 mM Ca^2+^. A single-channel trace with two active channels shows that the open probability does not decrease in the Ca^2+^ solution. Dashed lines indicate zoom in of a segment of the trace. All traces represent more than five independent patches.

Each subunit of MthK is composed of a short cytoplasmic N terminus of 18 amino acid residues followed by a 2-TM pore-forming domain. A RCK domain of approximately 220 residues is covalently linked to the C terminus of the second TM through a linker of 18 residues (Figure 1A, left). On the basis of the crystal structure of MthK (M107I mutant), four copies of a separately expressed, soluble RCK domain have been proposed to interact with the four membrane-tethered RCK domains in a pairwise manner to form a “gating ring” complex. Binding of Ca^2+^ to the domains extends the diameter of the ring and thus accounts for the activation gating [9,10]. Later on, it was discovered that besides Ca^2+^, MthK is also regulated by the pH of the cytoplasmic side such that the channel becomes completely inactivated at a pH value below 6.0 [11,12]. This acid inactivation has been proposed to be modulated by disassociation of a high-order RCK oligomer into dimers as demonstrated by both structural and biochemical analyses on the isolated RCK domain [10–13].

More recently, we discovered that the macroscopic current of MthK declines spontaneously after Ca^2+^ activation on a timescale of seconds, indicating the channel undergoes a process called desensitization [11]. This set of experiments was carried out by directly patch clamping the MthK channels expressed in enlarged E. coli membrane. However, the purified MthK channels studied in black lipid membrane (BLM) has not been observed to undergo desensitization [12,14,15]. This inconsistent observation raised a question as to whether this unique desensitization phenomenon observed in the enlarged E. coli system is an intrinsic molecular property or an experimental artifact. Besides the difference in the desensitization phenomenon, another set of inconsistent results was also observed from MthK studied by these two different recording systems. Single-channel analysis of purified MthK in the BLM system has shown that the open probability of MthK is drastically increased when the pH of the cytoplasmic side is raised to 8.0 and above, even without Ca^2+^ [12]. A gating ring model has been proposed to explain this alkaline-activation phenomenon in which alkaline pH extends the diameter of the octomeric gating ring in the absence of Ca^2+^ to open the channel gate [12]. However, this alkaline activation was not observed from the MthK studied in the enlarged E. coli system. Instead, the macroscopic currents show that MthK continues to require Ca^2+^ for activation followed by subsequent desensitization at alkaline pH up to 8.5 [11]. Additionally, our biochemical analysis of the isolated RCK domain at pH values higher than 8.0 has shown that the domain is predominantly monomeric when Ca^2+^ is absent [11]. This biochemical observation is also inconsistent with the proposed gating ring model made up by a stable octomeric RCK assembly [12].

In this report, we first demonstrate that the desensitization phenomenon is indeed intrinsic to the MthK channel, and the gating process is controlled by the cytoplasmic N-terminal 17 residues. By mutational and ^1^H NMR analyses, we further demonstrate that the desensitization gating mechanism may involve interactions between the N-terminal desensitization domain (DD) and C-terminal RCK domain. Additionally, we show that the desensitization and acid-inactivation gatings are controlled by distinct parts of the channel, and the processes are mutually independent. Finally, we demonstrate that MthK requires Ca^2+^ for activation in all ranges of pH as high as pH 9.0, the pH at which the isolated RCK domain undergoes a monomer-to-oligomer conversion by the presence of Ca^2+^.

## Results

### Desensitization of MthK Involves Its Cytoplasmic N Terminus

The MthK channels studied in this work were expressed in giant E. coli spheroplasts. Patch clamping with inside-out membrane patches was coupled with a rapid perfusion system to study the time-dependent channel kinetics in a timescale of milliseconds. Patches containing approximately 100–600 active wild-type MthK channels were usually observed when the giant spheroplasts were prepared with IPTG (isopropyl β-d-1-thiogalactopyranoside) treatment to promote the expression. This has allowed us to study the macroscopic behavior of the channel [11]. MthK has been shown to be activated by millimolar concentrations of Ca^2+^ with a half-effective concentration around 8.5 mM at pH 7.5 and −50 mV [11]. When activated by 20 mM or an excessive amount (100 mM) of Ca^2+^, the wild-type MthK current shows spontaneous decay within seconds, indicating that the channels undergo desensitization (Figure 1A, left and middle traces, respectively). Single-channel recording from giant spheroplasts without IPTG treatment reveals that the open probability of wild-type MthK decreases during the extended Ca^2+^ perfusion (Figure 1A, right trace).

In an attempt to test the functionality of MthK after being fused with the Mistic protein of Bacillus subtilis [16] to its N terminus (see Materials and Methods), we found that this Mistic-MthK chimera does not desensitize to Ca^2+^ at either 20 or 100 mM (Figure 1B, left and middle traces, respectively), though the number of active channels in an excised patch is drastically reduced. To test whether the disappearance of MthK desensitization is specific to the N-terminally fused protein, the Mistic protein was replaced with a 33-residue peptide containing a nona-histidine tag (Materials and Methods). Interestingly, the resulting 9xHis-MthK chimera also does not undergo desensitization (Figure 1C). These observations led us to hypothesize that the N terminus of MthK may be involved in the desensitization process in the wild-type channel.

To test this hypothesis, the entire cytoplasmic N terminus of MthK, from Val2 to Lys17, was deleted for examination. The macroscopic current of this Δ2–17 MthK channel shows rapid Ca^2+^ activation as the wild-type channel does, but the current does not decline during sustained Ca^2+^ perfusion at either 20 or 100 mM (Figure 1D, left and middle traces, respectively). The channel open probability also does not decrease during the extended Ca^2+^ perfusion (Figure 1D, right trace), indicating that the desensitization process is completely abolished in the Δ2–17 MthK. Therefore, we conclude that the short N terminus of MthK is required for desensitization.

### Synthetic N-Terminal Peptide Restores the Desensitization to Δ2–17 MthK In Trans

**Figure 2:**
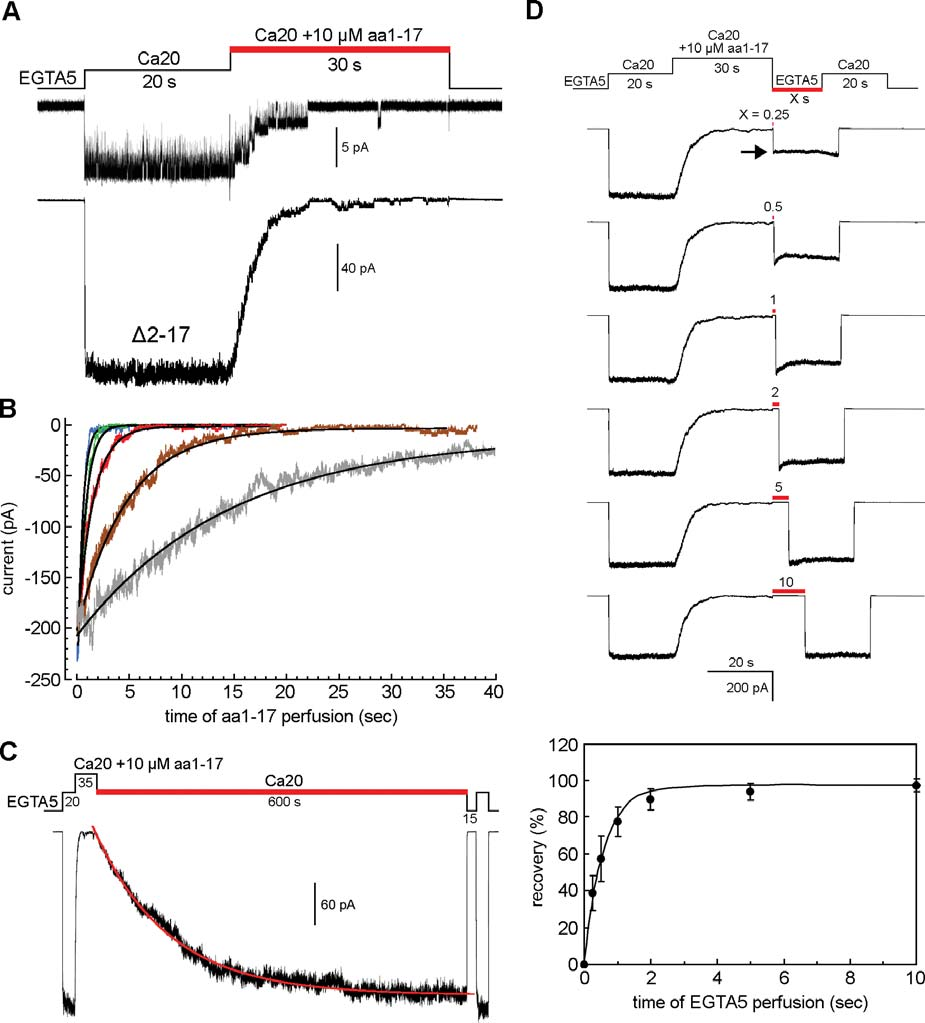
Effects of Synthetic N-Terminal 17-Residue Peptide on the Δ2–17 MthK Channels (A) A single-channel trace (top) shows that the channel open probability decreases when 10 μM synthetic peptide (aa1–17) was added to the perfusate (red bar). Bottom, a macroscopic trace shows that the Ca^2+^-activated current decays during the aa1–17 perfusion. The results indicate that the synthetic aa1–17 peptide is able to restore the desensitization phenomenon in trans. (B) Overlapped traces of the peptide-induced desensitization at different peptide concentrations. Smooth curves indicate single exponential fitting. Gray trace is 3 μM, which has a time constant of 14.75 ± 2.77 s; brown, 10 μM, 4.23 ± 1.34 s; red, 30 μM, 1.47 ± 0.34 s; green, 100 μM, 0.51 ± 0.13 s; blue, 1,000 μM, 0.37 ± 0.04 s (mean ± SD, *n* = 9–15). (C) A recording episode to determine the rate of recovery of the peptide desensitized channels back to the open state (red bar). The smooth curve shows single exponential fitting. The time constant is 135 ± 20 s (mean ± SD, *n* = 8). (D) Rate of recovery of the peptide desensitized channels back to the closed state (red bar). The time constant is 0.59 s (bottom panel, SDs are shown, *n* = 5).

The deletion experiment described above is reminiscent of the “ball” for the N-type inactivation in the mammalian *Shaker* K^+^ channels [17]. To test the idea, an artificial aa1–17 peptide, corresponding to the first 17 residues of MthK, was synthesized and added to the perfusate to test its effect on the Δ2–17 MthK. To this end, the Δ2–17 MthK channels in an excised inside-out patch were first activated by 20 mM Ca^2+^ (Ca20 solution) for 20 s (Figure 2A). The synthetic peptide was then added to the cytoplasmic side by stepping the perfusate to the same Ca^2+^ solution with an additional 10 μM aa1–17 peptide (Figure 2A, red bar). Surprisingly, the addition of the aa1–17 peptide drastically reduces the open probability of Δ2–17 MthK at the single-channel level (Figure 2A, upper trace), and causes the macroscopic current to decay down to zero in about 30 s (Figure 2A, bottom trace). These results show that the synthetic aa1–17 peptide is able to inhibit the activity of Δ2–17 MthK in a way similar to the desensitization process of the wild-type channel. Note that to distinguish this inhibitory process by the synthetic aa1–17 peptide versus the desensitization process by the natural N terminal DD in wild-type channel, we will refer to the inhibitory process as peptide desensitization.

The dose response of the peptide desensitization was examined between 3 to 1,000 μM. The time courses of the peptide desensitization can be fitted with single exponentials (Figure 2B). We found that at 10 μM, the rate of peptide desensitization (τ = 4.23 ± 1.34 s; Figure 2B, brown trace) is close to that of the wild-type MthK desensitization (5.7 ± 0.6 s; Figure 1A), which can be regarded as the virtually local concentration of the native DD in wild-type MthK. At concentrations above 100 μM, the time constant reaches its limit of around 0.3–0.5 s (Figure 2B, green and blue traces). The inhibition of the channel activity by the synthetic peptide is not permanent. The peptide-desensitized channels can be slowly, but readily, returned back to the open state after removing the peptide from the perfusate with a time constant of approximately 135 s (Figure 2C, red lines).

The rate of recovery back to the closed state was determined by simultaneously removing the Ca^2+^ and peptide using a two-activation protocol. As shown in Figure 2D, the Δ2–17 MthK channels in an excised patch were first activated by 20 mM Ca^2+^ for 20 s to determine the maximal activatable current before adding 10 μM aa1–17 peptide to fully desensitize the channels. The peptide and Ca^2+^ were then withdrawn simultaneously for various lengths of time (Figure 2D, red bar) to allow the peptide-desensitized channels to recover back to the closed state. The number of channels that have recovered to the closed state was determined by a second Ca^2+^ activation (Figure 2D, arrow). By comparing the amplitudes of the two Ca^2+^-activated peaks, we found that the time course of the recovery has a time constant of approximately 0.59 s (Figure 2D, bottom panel), which is about 200-fold faster than that of the recovery of wild-type MthK from the desensitized state back to the closed state (τ ∼110 s [11]). Since the DD in wild-type MthK is membrane tethered at the N terminus, the much faster recovery of the peptide-desensitized Δ2–17 MthK to the closed state can be partly explained by an entropic gain of the freely diffusing aa1–17 peptide.

### The Initial Hydrophobic Segment of the N Terminus Is Important for Desensitization

**Figure 3:**
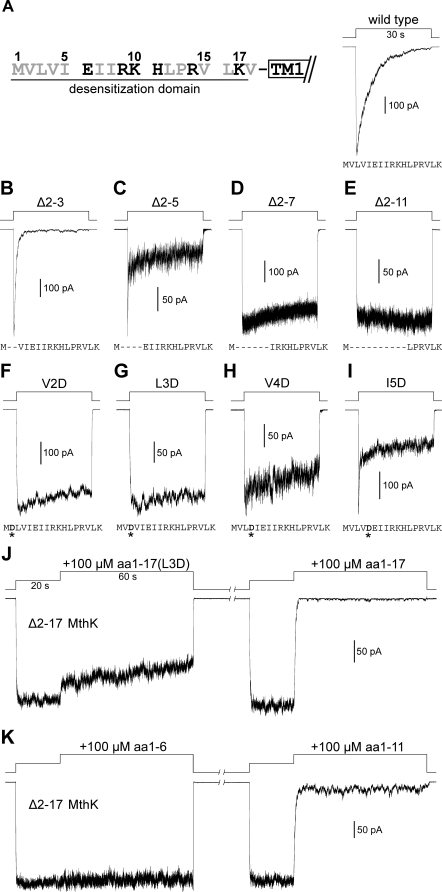
Mutational Analyses at the Cytoplasmic N-Terminus of MthK. Activation was done by stepping the perfusate from EGTA to Ca20 solutions at pH 7.5. (A) Left, amino acid sequence of the N-terminal desensitization domain (DD) of MthK. Hydrophobic residues are shown in gray. Right, a typical macroscopic trace of wild-type MthK. (B–E) Traces of sequential N-terminal deletion mutants show that truncations of DD result in significantly altered channel gating properties. (F–I) Traces of single-point mutations (asterisks) at the initial hydrophobic residues of the DD show that introducing a charged aspartate residue to disrupt the initial hydrophobicity drastically alters the gating profile. (J) Traces recorded from the same patch show the effects of 100 μM aa1–17(L3D) peptide (left trace) and aa1–17 peptide (right trace) on open Δ2–17 MthK channels. (K) Traces from the same patch, containing Δ2–17 MthK channels, show the effects of the aa1–6 (left trace) and aa1–11 (right trace) peptides. Typical traces are shown, representing four to six independent patches for (B–K).

The results thus far demonstrate that the cytoplasmic N-terminal 17 residues of MthK alone form a complete movable module, which can be deleted and added in trans to abolish or reconstitute the desensitization phenomenon, respectively. We then refer to it as the desensitization domain, DD (Figure 3A). In the original X-ray structure, the N-terminal 18 residues of MthK were not resolved in the structural model (Protein Data Bank [PDB] code 1LNQ [9]). Therefore, to define the residues or regions within DD that are important for the desensitization process, we performed mutational analyses within the DD by deletion and point mutation. In a series of sequential deletion, we found that the deletions within the first 11 residues often result in mutant channels with significantly altered gating properties. For examples, the deletion of two residues, from Val2 to Leu3 (Δ2–3), increases the rate of desensitization (τ = 995 ± 82 ms, *n* = 5; Figure 3B), whereas the deletion of four residues, from Val2 to Ile5 (Δ2–5), results in an activation spike followed by a further slow current decay (Figure 3C). Deletion of six residues, from Val2 to Ile7 (Δ2–7), results in partial desensitization (Figure 3D), and deletion of ten residues (Δ2–11) completely removes the desensitization process (Figure 3E).

The drastic effects of the N-terminal deletions on the gating profile led us to hypothesize that the initial hydrophobic segment, containing Met1, Val2, Leu3, Val4, and Ile5, may be important for desensitization. These hydrophobic residues were then replaced individually by an aspartate (D) reside to test the idea. Interestingly, introducing the charged residue at the second (V2D) or third (L3D) position removes the desensitization process almost completely (Figure 3F and 3G, respectively). At the fourth (V4D) and fifth (I5D) positions, the charged residue also significantly alters the gating property. (Figure 3H and 3I, respectively). The effect of the charged residue at the initial hydrophobic segment was also tested with a synthetic N-terminal peptide, containing a L3D mutation (aa1–17(L3D) peptide). When tested at 100 μM, the mutant aa1–17(L3D) peptide is much less effective to inhibit the Δ2–17 MthK activity (Figure 3J, left trace) than the wild-type aa1–17 peptide treated subsequently on the same patch (Figure 3J, right).

Since the disruption of the initial hydrophobicity has a profound effect on the desensitization process, we then went on to test whether this hydrophobic segment alone is able to inhibit the Δ2–17 MthK activity. Interestingly, this hydrophobic segment (aa1–6 peptide), when applied at 100 μM, has almost no inhibitory effect on the Δ2–17 MthK channels (Figure 3K, left trace). However, the longer aa1–11 peptide is able to inhibit the current as effectively (Figure 3K, right) as the aa1–17 peptide does (Figure 3J, right), but with a bit higher residual activity at the steady state.

### The Desensitization Domain Interacts with Isolated RCK Domain

**Figure 4:**
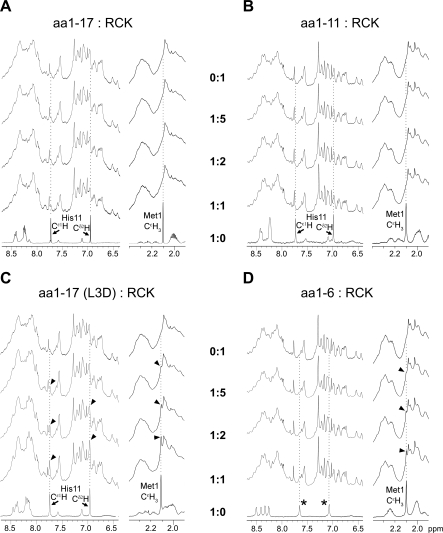
The 700-MHz ^1^H NMR Spectra of Isolated RCK Domain Titration by Synthetic Peptides The regions of 8.7…6.4 ppm and 2.4…1.9 ppm of the spectra are shown. The spectrum of the RCK domain without peptide is shown in the top rows; spectra of free peptides are shown in the bottom rows. Characteristic peaks are traced with the dotted lines. (A and B) Isolated RCK protein titrated with aa1–17 and aa1–11 peptides. The three characteristic peaks of the peptides disappeared when mixed with the RCK protein. (C and D) Isolated RCK protein titrated with the mutant aa1–17(L3D) and aa1–6 peptides. The characteristic peptide peaks are clearly detected in the peptide-RCK mixtures (arrowheads). Asterisks indicate peaks from the protons of the C-terminal amide group.

If the N-terminal DD of MthK is the primary structural determinant of the desensitization process, how does it render the channel into the desensitized state? As revealed in the crystal structure, the closest distance between Pro19 (the N terminus of TM1) and the membrane-facing side of the RCK domain is about 8 Å (PDB code 1LNQ). The physical proximity of the N- and C-terminal domains suggests that the DD may interact with RCK domain. To probe any possible interactions between these two domains in a sequence-specific manner, we used ^1^H NMR spectroscopy to analyze the behavior of synthetic DD peptides in response to the presence of isolated RCK domain in pH 7.5 solution. To this end, the aa1–17, aa1–17(L3D), aa1–11, and aa1–6 peptides, which have been functionally tested on the Δ2–17 MthK channel above, were analyzed individually by titrating the isolated RCK protein at various molar ratios (Figure 4). As shown in the bottom 1:0 traces of Figure 4A, 4B, and 4C, the aa1–17, aa1–11, and aa1–17(L3D) peptides give three distinct narrow peaks, corresponding to the C^ɛ1^H (7.73 ppm) and C^δ2^H (6.96…6.98 ppm) ring protons of the His11, and to the C^ɛ^H_3_–protons (2.10 ppm) of the Met1. With the aa1–6 peptide, the peak of C^ɛ^H_3_ protons of the Met1 was also observed at the same position (Figure 4D, bottom-right trace) together with two other peaks from the protons of the C-terminal amide group (Figure 4D, asterisks). These well-resolved, narrow peaks indicate that the peptides are in a rapidly tumbling state, corresponding to an unbounded form in the solution. If strong physical interactions between the peptide and the slowly rotating RCK protein occur, disappearance or displacement of these characteristic peaks will be observed. This is due to significant line broadening or shift, caused by a much shorter *t*_2_ relaxation time of the RCK protein or changes in these protons' local environment, respectively [18].

During the titration of RCK protein with peptides, significant differences in the behaviors of the four peptides were observed. For the aa1–17 peptide, the three characteristic peaks disappear at [aa1–17]:[RCK] ratios of 1:5 and 1:2 (Figure 4A, second and third rows, respectively). At 1:1 ratio, broadened and lowered intensity peaks of His11 ring protons were detected (Figure 4A, fourth row, left trace), but not the peak from Met1 (right trace). The similar disappearance of the peaks was also observed from the aa1–11 peptide (Figure 4B). Conversely, for the aa1–17(L3D) peptide, all three characteristic peaks were clearly detectable at the same positions, starting from 1:5 to 1:1 ratios (Figure 4B, arrow heads). For the aa1–6 peptide, the peak from Met1 was also detectable at the three titration ratios (Figure 4D, arrow heads). The disappearance of the characteristic peaks of the aa1–17 and aa1–11 peptides, but not those of the aa1–17(L3D) and aa1–6 ones, indicates that the aa1–17 and aa1–11 peptides have stronger interaction with the isolated RCK protein than the aa1–17(L3D) and aa1–6 peptides do in a residue-specific manner.

### Closed Δ2–17 MthK Can Be Desensitized by a High Concentration of aa1–17 Peptide

**Figure 5:**
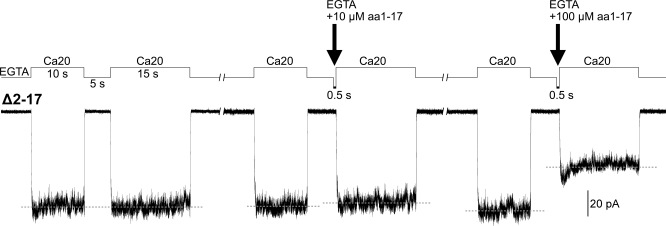
Behavior of Δ2–17 MthK upon the Perfusion of Synthetic aa1–17 Peptide to the Cytoplasmic Side of the Patch When the Channels Are in the Closed State Left, trace of two Ca^2+^-activation peaks shows the channels in the excised patch, after being activated by the first Ca20 perfusion, are able to return fully to the closed state during the 5-s EGTA perfusion. Dashed lines indicate the average amplitude of the peaks. Middle and right, traces show the effects of adding 10 and 100 μM DD peptide (arrows) to the closed channels, respectively. Traces are from a single patch, representing five independent patches.

In Figure 2, we showed that applying the DD peptide while the Δ2–17 MthK channels are in the open state can inhibit the channel activity. The decrease in the macroscopic current may be due to either open pore blockage, similar to the “ball-and-chain” model of the *Shaker* K^+^ channels, or an allosteric blockage, possibly through an interaction between RCK and DD. To delineate between these two possible mechanisms, we tested the effect of the DD peptide on the closed channels by adding the peptide to the EGTA solution. To this end, the Δ2–17 MthK channels in an excised patch were first activated twice with a 5-s interval in-between to be certain that the 5-s EGTA perfusion, after the first Ca^2+^ activation, is able to completely return the channels to the closed state (Figure 5, left trace). Ten or 100 μM synthetic DD peptide in EGTA solution was then added to the perfusate, after the 5-s EGTA perfusion, for 0.5 s prior to the second Ca^2+^ activation (Figure 5, left and right arrows, respectively). Since the channels have fully returned to the closed state during the 5-s EGTA perfusion, applying DD peptide afterward allows examination of the peptide effect on channels that are in the closed state. Interestingly, by comparing the amplitude of the two Ca^2+^-activation peaks, we found that the perfusion of 100 μM DD peptide is able to inhibit about 40% (43.3 ± 13.9%, *n* = 5) of the closed channels (Figure 5, right trace), whereas 10 μM DD peptide has little effect on the closed channel (5.1 ± 3.6% inhibited, *n* = 5; Figure 5, middle trace). Since no channel opening was observed during the 0.5 s of peptide perfusion, these results demonstrate that the synthetic aa1–17 peptide, when applied at high concentration, is able to shift the equilibrium of the Δ2–17 MthK channel directly from the closed to the peptide-desensitized state.

### pH Effects on Acid-Inactivation Gating and Activation Gating

**Figure 6:**
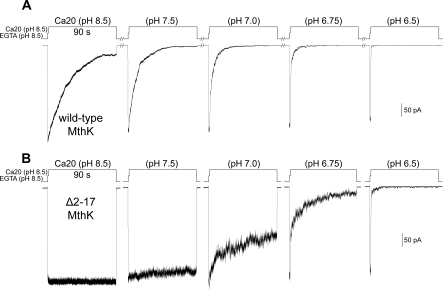
Behavior of Wild-Type and Δ2–17 MthK in Response to the pH of the Cytoplasmic Side The channels were activated by stepping the perfusate from EGTA (pH 8.5) to Ca20 (pH 8.5) solutions for 1 s, and then to Ca20 solution at different pH values for 90 s. (A) Traces of wild-type MthK currents recorded from the same patch, representing five independent patches. After each activation episode, the patch was perfused with EGTA (pH 8.5) solution for more than 8 min for the channels to recover back to the closed state. (B) Traces of Δ2–17 MthK show that the currents were inactivated by neutral to slightly acidic pH. Ensemble currents of three independent patches are shown.

Our previous analysis has shown that the rate of macroscopic decay of the wild-type MthK current depends on the pH of the Ca^2+^ solution (Figure 6A [11]). Since the desensitization process can now be completely removed by deleting the DD, we also examined the pH response of the Δ2–17 MthK to see whether there is a correlation between the desensitization and the acid-inactivation processes. As shown in Figure 6B, at pH above 7.5, the macroscopic currents of Δ2–17 MthK show little decline (Figure 6B, left two traces), whereas dramatic decay in the macroscopic currents starts when the pH is shifted to 7.0 or lower (Figure 6B, right 3 traces), indicating that the pH inactivation is insignificant at pH above 8.5 and maximized at pH below 6.5. This result is similar to that determined from wild-type MthK [11], suggesting that the deletion of DD has little effect on the acid-inactivation gating process, and the two inactivation processes have no synergy effect.

**Figure 7:**
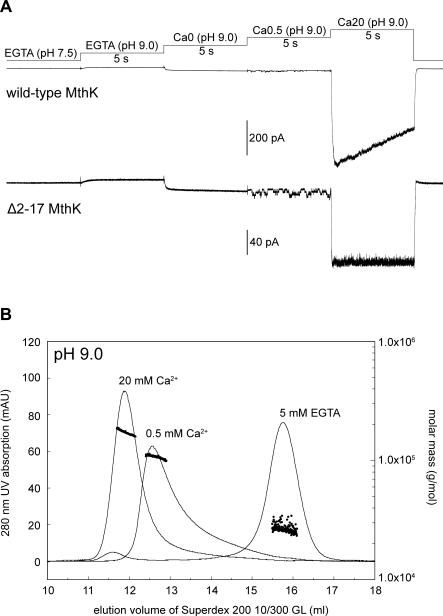
Behavior of MthK and the Isolated RCK Domain in Response to Ca^2+^ at pH 9.0 (A) Representative macroscopic traces of wild-type MthK (upper trace) and Δ2–17 MthK (bottom trace) show that stepping the perfusate from EGTA (pH 7.5) to EGTA (pH 9.0) then to Ca0 (pH9.0) solution does not activate the channels. Activation of MthK at pH 9.0 still requires sub- to millimolar concentrations of Ca^2+^ (last 2 steppings). Digitized at 2.5 kHz and filtered at 1 kHz. Ca0, Ca0.5, and Ca20 solutions contain (in mM) 10 Tris-HCl (pH 9.0), 150 KCl, 500 sucrose, and 0, 0.5, or 20 CaCl_2_, respectively. (*n* = 3 patches). (B) Oligomeric states of the isolated RCK domain at pH 9.0 in the presence of EGTA or Ca^2+^, determined by size-exclusion chromatography (smooth lines) and static light scattering (dots). The retention volumes for each of the corresponding peaks are 11.9 ml (20 mM Ca^2+^), 12.6 ml (0.5 mM Ca^2+^), and 15.8 ml (5 mM EGTA). Molar masses for each of the corresponding peaks are 170 ± 10 kDa (20 mM Ca^2+^), 106 ± 4 kDa (0.5 mM Ca^2+^), and 26.5 ± 2.3 kDa (5 mM EGTA).

Single-channel analysis on purified MthK in BLM has shown that the channel open probability is drastically increased when the pH of the cytoplasmic side is above 8.0, even in the absence of Ca^2+^ [12]. We also examined this alkaline pH effect on both wild-type and Δ2–17 MthK in E. coli membrane. Contrary to the BLM data, our analyses show that the activation of either wild-type or Δ2–17 MthK at pH values as high as 9.0 still requires the presence of at least submillimolar concentration of Ca^2+^ (Figure 7A, upper and lower traces, respectively). When examined at pH 9.0, the oligomeric state of the isolated RCK domain is predominately monomeric in EGTA solution and multimeric in Ca^2+^ solutions (Figure 7B), much the same as what we previously observed at pH 8.5 [11]. The gating behavior of MthK at extreme alkaline pH values examined in E. coli membrane correlates well with the solution behavior of the isolated RCK domain, which supports the idea that the activation gating of MthK is controlled by oligomeric RCK conversion [11].

## Discussion

### Functional Analyses of MthK

Previous studies of MthK were carried out by purifying the channel proteins and reconstituting them in BLM [12,14,15]. Although this method ensures that the activities observed originate from the pure proteins, the BLM system cannot readily resolve the rapid ligand-gating kinetics because of the time required for chamber perfusion. Therefore, the gating properties previously studied in BLM may not reflect those of activation gating [19]. Biochemical analyses of the isolated RCK domain of MthK have shown that the domain is able to form various oligomeric states, including 1-mer, 2-mer, 4-mer, 6-mer, and 8-mer, depending on the pH and Ca^2+^ [10,11,13]. In this study, we propose that the synthetic N-terminal DD induces desensitization by interacting with the RCK domain (below). Based on these observations, it is possible that the separately expressed free RCK protein, which has been shown to be copurified with the full-length MthK [9,14], can bind to either the N-terminal DD or the membrane-tethered C-terminal RCK domain. Although the native form of the DD-RCK or RCK-RCK interaction is unclear, these interactions could possibly be altered during the purification and reconstitution processes. For instance, the crystal structure of the MthK (M107I mutant) shows that the two channels interact with each other through the member-tethered RCK domains after being extracted and purified [9]. Therefore, whether the conformation of the reconstituted MthK in the BLM system remains the same as those in the cell membrane needs to be established.

To avoid possible alternations in the natural conformation, we have expressed MthK in the membrane of giant E. coli spheroplasts for direct patch clamp [11]. The desensitization property was discovered when the excised patches were bathed in a stream of perfusate that can be switched within tens of milliseconds [11]. The E. coli patch-clamp method forgoes the simplicity of BLM reconstitution but provide a natural setting to examine the channel without altering the native RCK assembly. However, a logical possibility exists that a protein native to E. coli may interact with the MthK protein from the cytoplasm, causing it to desensitize. In this study, we addressed this question by showing that the desensitization phenomenon of MthK can be removed by deleting its N-terminal DD and can be re-established by a synthetic DD peptide, and its shorter variants, added in trans. These results are consistent with the conclusion that the desensitization process is an intrinsic molecular property. To further characterize how the DD and RCK domains modulate the gating of MthK, reconstituting purified channels into an artificial liposome for patch clamp may provide a more advanced system to circumvent possible heterologous interactions in giant E. coli system, as well as to resolve rapid kinetics [20–22].

### Mechanism of MthK Desensitization

Our initial observations had reminisced the works of the N-type ball-and-chain inactivation model in the voltage-gated *Shaker* K^+^ (Kv) channels [17]. In *Shaker*-type Kv channels, the N-type inactivation was restored to inactivation peptide (IP)-truncated channels by a synthetic IP segment of approximately 20 amino acids located at the N-terminal end of the *Shaker* channel subunits [23,24]. The current model for *Shaker* inactivation is that the IP ball, as an unfolded chain, reaches the intracellular cavity of the pore through the lateral opening between T1 and the TM domain of the channel. In this model, a single IP acts like a quaternary ammonium channel blocker and can access and physically occlude the central ion pathway of an open channel [25–33].

In MthK, the DD, which is about the size of the IP ball, is directly attached to the MthK channel body without a “chain” as seen in the *Shaker* channels. Therefore, does the DD desensitize MthK by reaching the intracellular cavity to block the pore, or by interacting with other parts of the channel to cause allosteric blockage? Our analyses using the synthetic DD peptide showed that the rate of peptide desensitization varies between subseconds to tens of seconds, depending on the peptide concentration. And the recovery of the peptide-desensitized channel depends on Ca^2+^, such that the recovery is much faster when Ca^2+^ is removed than when present. This kinetic behavior of DD peptide blockage is quite different from that of the TBA (tetrabutylammonium) pore blockage. When examined between 0.01 and 10 mM, the rates of TBA blockage and recovery on open Δ2–17 MthK channels are both around tens of milliseconds (unpublished data). Since the quaternary ammonium compound is known to block K^+^ channels by directly plugging into the intracellular cavity of the pore [31], the much slower rates of DD peptide blockage suggest that the blocking mechanism may not simply be the same as the TBA pore blocker. Consistent with this conclusion is the observations that the shorter aa1–6 peptide has almost no blocking effect, and the aa1–11 peptide is not able to fully block the Δ2–17 MthK channels as the aa1–17 peptide does at steady state. These results indicate that the initial hydrophobic segment of the DD alone is not able to act like the quaternary ammonium pore blocker. Instead, the subsequent residues, from Ile7 to Lys17, also play a role to complete the gating modulation. Furthermore, in Figure 5, we showed that perfusion of DD peptide at substantially high concentration to the cytoplasmic side of Δ2–17 MthK can desensitize the channels without opening. This result indicates that the binding site for the DD peptide is accessible in the closed channel, and the DD blockage is rather allosteric.

In the ^1^H NMR analyses, we demonstrated that the aa1–17 and aa1–11 peptides, but not the mutant aa1–17(L3D) or aa1–6 peptide, directly interact with the isolated RCK domain. These binding results correlate well with the functional analyses in which the aa1–17 and aa1–11 peptides, but not the mutant aa1–17(L3D) or aa1–6 peptide, are able to inhibit the K^+^ current. This direct correlation between protein binding and channel blockage suggests that the physical interaction of the synthetic DD peptide to the RCK domain is required to cause the peptide-desensitization. Although the results of peptide desensitization should be interpreted with caution to surmise the native desensitization mechanism, it is plausible that the N-terminal DD in the wild-type MthK interacts with the C-terminal RCK domain in the similar way to render the channel into the desensitized state after Ca^2+^ activation. Further functional characterizations of the natural and peptide desensitization are required to compare the differences and similarities of these two mechanisms. Note that this set of experiments highlights that an interaction between the N-terminal DD and C-terminal RCK domain may be responsible for the desensitization gating process; however, it does not exclude additional interaction of DD with other parts of the channel, including the transmembrane pore region. Given that the structure of DD is not resolved in the crystal structure of MthK (M107I), further elucidating the detailed DD-RCK interaction of the entire MthK structure at atomic scale may help understanding how the interactions participate in the gating process.

Results in this report together with previous studies of others provide a converging view that the gating mechanisms of activation, of activation-coupled desensitization and of the acid-induced inactivation are largely mediated by conformational changes in the RCK domain, namely the desensitization by DD and the acid inactivation by RCK disassembly into dimers at acidic pH [11–13]. For activation gating, however, it remains to be better understood how Ca^2+^-triggered conformational change of the RCK domains results in channel opening. In conclusion, these dynamic Ca^2+^-, pH-, and DD-dependent conformational changes of RCK domain underlie the mechanistic basis of MthK gating.

## Materials and Methods

### Molecular biology.

The gene of wild-type MthK was cloned into the pB11d vector between the NcoI and XhoI sites behind a LacUV5 promoter. The N-terminal mutants were created by designing the 5′ PCR mutant primer with a restriction cutting site compatible with NcoI for ligating into the vector. All the mutations were confirmed by DNA sequencing. The Mistic-MthK and 9xHis-MthK chimeras were made by cloning the MthK ORF into a Gateway-adapted Mistic-containing pMIS4 and a Gateway-adapted pHis9 vector [11], respectively. The amino acid sequences before the Methionine1 of MthK in the 9xHis-MthK chimera is MKHHHHHHHHHGGLESTSLYKKAGSLVPRGSGS (33 residues). Mistic is a “membrane-integrating” protein from B. subtilis. It was originally discovered for its ability to increase the expression of eukaryotic membrane proteins in E. coli when it is fused to the N terminus [16,34]. We originally made the Mistic-MthK chimera to study potential effects of Mistic on the functionality of its MthK cargo.

### Electrophysiology.

The preparation of giant E. coli spheroplasts and the patch-clamp recordings were performed following the protocol previously described [11,35] with minor modifications. In brief, the plasmids containing wild-type or mutant MthK were transformed in FRAG1 (*Δkch*) strain [36] for expression. The Gateway-adapted plasmids, containing the chimeras, were transformed in the BL21Star(DE3) strain (Invitrogen). A fresh single colony was inoculated in 5 ml of modified LB medium (10 g/l tryptone, 5 g/l yeast extract, and 5 g/l NaCl), supplemented with an antibiotic to maintain the plasmid. The culture was incubated at 250 rpm, 37 °C, until the optical density at 600 nm (OD_600_) reaches approximately 0.3, and then diluted 10-fold into a prewarmed modified LB medium, supplemented with the antibiotic and 60 μg/ml cephalexin to block cell fission. For macroscopic recordings, 0.5 mM IPTG was added to the culture after 2 h of incubation to promote the gene expression for 1.5 h. For single-channel recordings, the culture was incubated for 4 h at 250 rpm, 37 °C without adding IPTG. The expression from the basal leakage of the LacUV5 and T7 promoters allows us to obtain patches containing fewer than ten channels (Figure 1, right traces). The filamentous cells were harvested in a 1.5-ml Eppendorf tube by centrifugation and then resuspended with 500 μl of 0.8 M sucrose. Thirty microliters of 1 M Tris-HCl (pH 8.0), 24 μl of 0.5 mg/ml lysozyme, 6 μl of 5 mg/ml DNase, and 6 μl of 125 mM EDTA-NaOH (pH 8.0) were added in sequence and mixed immediately in-between by inverting the tube a few times. After approximately 8 min of incubation at room temperature, 100 μl of Stop Solution (10 mM Tris-HCl [pH 8.0], 0.7 M sucrose, 20 mM MgCl_2_) was added to terminate the digestion. The spheroplasts were directly used for patch clamp or frozen at −80 °C for later use.

For all the patch-clamp recordings, the pipettes were filled with the Ca20 solution, containing (in mM) 10 Hepes-Tris (pH7.5), 500 sucrose, 150 KCl, and 20 CaCl_2_. The EGTA solution contains 10 Hepes-Tris (pH7.5), 500 sucrose, 150 KCl, 20 MgCl_2_, and 5 EGTA. The bath was filled with either the Ca20 or EGTA solution together with the giant spheroplasts for gigohm seal formation. (The 500 mM sucrose provides osmotic protection and prevents the giant spheroplast from bursting during the seal formation. For unknown reasons, the formation of the gigohm seal and stabilization of the gigohm seal during the prolonged experimental perfusion require the presence of millimolar concentration of either Ca^2+^ or Mg^2+^ at both sides of the membrane patch. Thus, 20 mM Ca^2+^ or Mg^2+^ was added in the pipette and the EGTA solutions, respectively.) A seal resistance of 3–5 GΩ was often reached. After being excised, the pipette tip was positioned in front of the opening of a single-walled, three-barrel glass tube (0.7-mm ID) of the SF-77B perfusion system (Warner Instruments). The perfusates were gravity fed, and the flow speed at the opening of the tubing was estimated to be approximately 0.5 cm/s. The speed of perfusate exchange (from the beginning of the stepping signal to the activation of channels) is approximately 61 ± 14 msec (*n* = 75). The excised membranes were held at −50 mV for all the recordings. Signals were amplified by an EPC7 Patch Clamp Amplifier (HEKA Instruments). The macroscopic currents were digitized at 1 kHz by a Digidata1322A digitizer (Axon Instruments) and filtered at 500 Hz by an in-line eight-pole Bessel filter (Frequency Devices) unless otherwise stated. Single-channel currents were digitized at 25 kHz and filtered at 5 kHz (further filtered at 1 kHz with a Clampfit 9 software for presentation). All statistics are shown as mean ± the standard deviation (SD).

The synthetic peptides, aa1–17 (MVLVIEIIRKHLPRVLK-[NH_2_]), aa1–17(L3D) (MV**D**VIEIIRKHLPRVLK-[NH_2_]), and aa1–6 (MVLVIE-[NH_2_]), were from Sigma-Genosys, and aa1–11 (MVLVIEIIRKH-[NH_2_]) was from Celtek Peptides. Stock solutions were prepared at 1–30 mM in water, and their actual concentrations were determined with a NMR using DSS (2,2-dimethyl-2-silapentane-5-sulfonic acid) as an internal standard. Before usage, the stocks were diluted into the perfusion solutions and the pH was retitrated to 7.5.

### RCK purification and ^1^H-NMR binding analyses.

Isolated RCK domain (M107-A336) for NMR study were purified from the soluble fraction of the cell lysate in which the wild-type MthK was expressed from the pQE70-MthK plasmid (a gift from R. MacKinnon) [9,13]. The purified RCK protein, after thrombin cleavage of the C-terminal His-tag, was dialyzed into 20 mM Tris-HCl (pH 7.5), 150 mM KCl, and 1 mM DTT and concentrated to approximately 200 μM for NMR analysis.

NMR spectra were recorded using Bruker Avance700 spectrometer equipped with CryoProbe. Spectra were recorded at 25 °C, with a 4-s relaxation delay. The initial peptide concentrations were verified using 0.1 mM DSS as a concentration standard. Chemical shifts were calibrated using DSS-Si-(CH_3_)_3_ signal as the internal standard at 0.0 ppm. For all peptide titration sets, NMR spectra were recorded for the following [peptide]:[RCK] ratios: 0:1, 1:5, 1:2, 1:1, and 1:0. Peptide solutions for titration were prepared using RCK dialysis buffer (20 mM Tris-HCl [pH 7.5], 150 mM KCl, and 1 mM DTT).

### RCK purification, size-exclusion chromatography, and static light scattering.

Isolated RCK domain (M107-A336) were expressed and purified as described previously [11]. The purified protein, after thrombin cleavage of the N-terminal 9xHis tag, was N-terminal sequenced, and its molecular weight (25.7 kDa) was confirmed by mass spectrum analysis. The protein was dialyzed into 20 Tris-KOH (pH 9.0), 150 K^+^(Cl^−^), 5 EGTA, and 1 DTT, and concentrated to approximately 5 mg/ml for injection. The protein was further dialyzed into 20 Tris-KCl (pH 9.0), 150 KCl, and 1 DTT to remove EGTA. Concentrated CaCl_2_ solution was added to the protein sample to the desired final concentration before injection into a Superdex 200 10/300 GL column. In-line static light scattering was performed as described previously [11].

## Abbreviations

^1^H NMR: proton nuclear magnetic resonance
BLM: black lipid membrane
DD: desensitization domain
IP: inactivation peptide
TM: transmembrane
